# Bayesian Prior Choice in IRT Estimation Using MCMC and Variational Bayes

**DOI:** 10.3389/fpsyg.2016.01422

**Published:** 2016-09-27

**Authors:** Prathiba Natesan, Ratna Nandakumar, Tom Minka, Jonathan D. Rubright

**Affiliations:** ^1^Department of Educational Psychology, University of North TexasDenton, TX, USA; ^2^School of Education, University of DelawareNewark, DE, USA; ^3^Microsoft Research CambridgeCambridge, UK; ^4^National Board of Medical ExaminersPhiladelphia, PA, USA

**Keywords:** Bayesian, item response theory, variational Bayesian, marginal maximum likelihood, Markov chain Monte Carlo

## Abstract

This study investigated the impact of three prior distributions: matched, standard vague, and hierarchical in Bayesian estimation parameter recovery in two and one parameter models. Two Bayesian estimation methods were utilized: Markov chain Monte Carlo (MCMC) and the relatively new, Variational Bayesian (VB). Conditional (CML) and Marginal Maximum Likelihood (MML) estimates were used as baseline methods for comparison. Vague priors produced large errors or convergence issues and are not recommended. For both MCMC and VB, the hierarchical and matched priors showed the lowest root mean squared errors (RMSEs) for ability estimates; RMSEs of difficulty estimates were similar across estimation methods. For the standard errors (SEs), MCMC-hierarchical displayed the largest values across most conditions. SEs from the VB estimation were among the lowest in all but one case. Overall, VB-hierarchical, VB-matched, and MCMC-matched performed best. VB with hierarchical priors are recommended in terms of their accuracy, and cost and (subsequently) time effectiveness.

## Introduction

Developing accurate parameter estimation methods is an important problem in item response theory (IRT). Currently, marginal maximum likelihood (MML) is the most widely used parameter estimation technique in IRT. However, advances in computational statistics have made Bayesian estimation, especially Markov Chain Monte Carlo (MCMC; Patz and Junker, 1999; Gelman et al., 2013) techniques, a plausible alternative for IRT parameter estimation. Two possible reasons for the lack of adoption of Bayesian inference are (1) MCMC runs much slower than MML and (2) it is not obvious how to choose appropriate priors. In this paper, we address both of these issues. To address computational efficiency, we suggest variational Bayesian (VB; Beal and Ghahramani, 2003) inference, which provides answers close to MCMC at a fraction of the time and cost. Both prior choice and the appropriateness of VB to IRT were investigated using simulation.

In general, Bayesian methods have advantages over traditional estimation approaches because their estimates are asymptotically distribution free and therefore depend less on the distribution of the data (Ansari and Jedidi, 2000). Bayesian methods overcome some drawbacks of MML, such as lack of efficiency in smaller samples, and inaccurate estimation of parameters with extreme response patterns (Lord, 1986; Baker and Kim, 2004). In particular, Bayesian methods have a potential advantage over MML in small samples and when the examinee ability distribution is not normal.

A main estimation difference between MML and Bayesian methods is the necessity of specifying priors to estimate the posterior distribution of parameters. In a Bayesian framework, prior specification allows for the systematic incorporation of previous information into the current estimation (Fox, 2010). Although the effect of priors on parameter estimates is quite minimal in large samples, priors can have a considerable impact in small samples. Therefore, appropriate prior choice is an important issue to be addressed when using these methods in the estimation of IRT models. However, a review of Bayesian procedures in the IRT literature shows that the use of priors has been largely inconsistent, leaving the field with little guidance on appropriate prior use. For instance, in estimating the parameters of a one-parameter logistic model (1-PL), Ghosh et al. (2000) used multivariate t-distribution priors for ability and flat priors for difficulty. Swaminathan and Gifford (1982) used a unit normal prior for ability and a hierarchical prior for difficulty, (*b* ~ *N*(μ, σ), μ ~ *uniform prior* and σ ~ *inverse χ*^2^ *with* ν = 10 *and λ* = 10, where μ and σ are the mean and standard deviation of the distribution from which the difficulty parameter *b* is drawn). Although Swaminathan and Gifford (1982) discouraged the use of extremely optimistic priors such as the unit normal distribution, or very diffuse priors with large variances, these continue to be used in the field (e.g., Patz and Junker, 1999; Kim, 2001; Fox, 2010). Gao and Chen (2005) studied four different prior specifications in estimating the difficulty parameters of a 3-PL model. However, all four prior distributions were relatively informative (beta distributions with *SD* < 0.9 and uniform distributions ranging from −3 to 3). Sheng (2010) compared the impact of prior variances specified using beta distributions on three-parameter normal Ogive model. She discouraged the use of very small prior variances for small samples. However, there is no guidance on how small is small enough. Furthermore, she suggested that the performance of hierarchical priors be tested for IRT models. Gelman et al. (2008) compared the performance of priors derived from the data for logistic regression models. However, they warned against the use of this method for small sample sizes. There is very little consensus about appropriate prior use in the estimation of IRT models. Parameter recovery using diffuse priors such as those used by Kim (2001) and hierarchical priors such as those used by Swaminathan and Gifford (1982) have not been compared.

Building upon the works of Swaminathan and Gifford (1982), Lord (1986), and Kim (2001), the purpose of this study was to investigate the choice of prior distributions in Bayesian estimation of one-parameter (1-PL) and two-parameter (2-PL) models. This study focuses on parameter recovery of Bayesian methods (both the traditional MCMC and the newer variational Bayesian) under different prior choices. Although some studies have compared the performance of MCMC with MML for graded response models (Kieftenbeld and Natesan, 2012), nominal response models (Wollack et al., 2002), and generalized graded unfolding models (Roberts and Thompson, 2011), the choice of priors and variational Bayesian (VB) estimation have not been studied for IRT models. In sum, no study has comprehensively compared the performance of vague and hierarchical priors for MCMC and VB estimates of IRT models.

Both the 1-PL and 2-PL IRT models are popular in applied practice. These models are easier to estimate and have lower sample size requirements than the 3-PL model, which includes a lower asymptote, pseudo-guessing parameter. These models are readily suitable for a wide range of applications such as dichotomously scored achievement test items, response time models, and voting preference models in political science. Since the variational Bayesian estimation method (VB) is relatively new and has not been investigated for any IRT models, these popular and comparatively simple IRT models are examined to evaluate prior choice and the use of a newer Bayesian estimation methodology.

In this study, parameter recovery of two Bayesian estimation techniques, MCMC and VB, was investigated for different prior distribution choices using simulated data. Conditional maximum likelihood (CML) and marginalized maximum likelihood (MML) were used as a baseline for comparison. CML is the most widely used estimation method for the 1-PL model, while MML is the most widely used estimation method for IRT models more generally.

## Parameter estimation

For the 2-PL model, the probability of a correct response *Y* for an individual *i* with ability level θ_*i*_ on item *k* with difficulty parameter *b*_*k*_ and discrimination parameter *a*_*k*_ is given as:

(1) $$\begin{array}{l} P\left(Y_{ik}=1|\theta_{i},b_{k},a_{k}\right)=\frac{\exp\left(a_{k}\left(\theta_{i}-b_{k}\right)\right)}{1+\exp\left(a_{k}\left(\theta_{i}-b_{k}\right)\right)} \end{array}$$

Equation (1) reduces to the 1-PL model when the discrimination parameter is constrained to a constant.

## Bayesian estimation

Bayesian estimation uses prior information about the characteristics of parameters and the conditional likelihood of the data given the model parameters to obtain the joint posterior density of the model parameters. In Equation (2) below, *f*(Ω|*X*) represents the joint posterior density of the model parameters given the data, *f*(*X*|Ω) represents the likelihood of the item response data given the model parameters (under conditions of local item independence^1^), and *f*(Ω) is the prior density of the model parameters:

(2) $$\begin{array}{l} f\left(\Omega |X\right)\propto \;f\left(X|\Omega \right)f\left(\Omega \right) \end{array}$$

Unlike MML which focuses on point estimates, Bayesian estimation focuses on the joint posterior distribution whose summary statistics yield extensive information about the parameters (e.g., credibility intervals which indicate probability that the true value is contained in this interval). Adaptability to complex models and restriction of estimates to reasonable ranges (e.g., discrimination parameter will not be infinite) are other advantages of Bayesian estimation (Fox, 2010). However, the choice of priors may introduce bias in the estimates, such as estimates being regressed toward the mean of the assigned prior, especially in small samples. Possible non-convergence of parameter estimates, specifying appropriate priors, and intensive computation are potential problems.

Prior specification may be the largest advantage, yet potentially the greatest drawback, of implementing Bayesian methods. The flexibility in specifying priors helps estimate complex sampling designs and dependency structures in Bayesian estimation (for more details refer Fox, 2010). Priors also allow the researcher to include information from previous research in a systematic manner. In cases where little is known about the population distribution, extra care is required in specifying the prior so that it expresses the uncertainty about the population without being too vague. The two Bayesian estimation techniques under study, MCMC and variational Bayesian, are briefly described next.

### MCMC estimation

MCMC uses the proportionality in Equation (2) to evaluate the relative likelihoods of parameter estimates. Ultimately, the goal of MCMC is to reproduce the *f*(Ω|*X*) distribution, which often cannot be determined analytically. Therefore, the characteristics of the distributions are determined by sampling enough observations from the posterior. The Gibbs sampler is one such technique that samples with respect to univariate conditional distributions of the model parameters (Geman and Geman, 1984; Gelfand and Smith, 1990). The Gibbs sampler uses conditional posterior distributions to obtain a chain of draws of the model parameters, **ω** = (ω_1_, …, ω_*P*_). The algorithm starts with initial values $\omega^{\left(0\right)}=({\omega_{1}}^{\left(0\right)},\;\ldots ,\;{\omega_{P}}^{\left(0\right)})$ then iteratively updates **ω**^(*t*−1)^ to **ω**^(*t*)^ by sampling as follows:

(3) $$\begin{array}{l} {\omega_{1}}^{\left(t\right)}\;\sim \;p\left(\omega_{1}|{\omega_{2}}^{\left(t-1\right)},\;\ldots ,\;{\omega_{P}}^{\left(t-1\right)},\;\text{Y}\right)\\ {\omega_{2}}^{\left(t\right)}\;\sim \;p\left(\omega_{2}|{\omega_{1}}^{\left(t\right)},{\omega_{3}}^{\left(t-1\right)},\;\ldots ,\;{\omega_{P}}^{\left(t-1\right)},\;\text{Y}\right)\\ \cdot \\ \cdot \\ \cdot \\ {\omega_{P}}^{\left(t\right)}\;\sim \;p\left(\omega_{P}|{\omega_{1}}^{\left(t\right)},\;\ldots ,\;{\omega_{P-1}}^{\left(t\right)},\;\text{Y}\right). \end{array}$$

The distribution of **ω**^(*t*)^ converges to the posterior distribution *p*(**ω**|***Y***). Usually, the influence of the initial values are allowed to “burn-in” by discarding the first *B* iterations in a chain (**ω**^(0)^, …, **ω**^(*T*)^) of length *T*. A point estimate $\hat{\omega }$ for a parameter ω is the posterior mean of the marginal posterior distribution *p*(ω|***Y***), which can be approximated by the mean of the samples as:

(4) $$\begin{array}{l} \hat{\omega }=\frac{1}{\left(T-B\right)}\sum_{t=B+1}^{T}\omega^{\left(t\right)}. \end{array}$$

### Variational bayesian estimation

VB is used to approximate intractable integrals by specifying a family of approximate distributions and then finding the member of this family that minimizes divergence to the true posterior (Bishop, 2006). An advantage of VB over MCMC is the reduction of computation by approximating the posterior with a simpler function, leading to faster estimation. A disadvantage may be some loss of accuracy because of this approximation. The present study investigated the extent of the loss of accuracy in exchange for faster estimation of 1-PL and 2-PL model parameters.

In the 1-PL model, the ability levels θ and difficulty parameters *b* are the unknown parameters, both of which have real values. Therefore, an approximating family in which the parameters were Gaussian and independent was chosen for the VB approach. The approximate distribution is

(5) $$\begin{array}{l} q\left(\theta ,b\right)=\underset{i}{\prod }q\left(\theta_{i}\right)\underset{k}{\prod }q\left(b_{k}\right), \end{array}$$

where *q*(θ_*i*_) denotes a Gaussian probability density function with two free parameters (a mean and variance) for each *i*, thus giving a point estimate plus uncertainty for each parameter. These means and variances are optimized by minimizing the Kullback–Leibler divergence *KL*(*q*(θ, *b*)||*p*(θ, *b*)). Kullback–Leibler divergence is a non-symmetric measure of the difference between the distributions *p* and *q* (KL, Kullback and Leibler, 1951). Here, *p*(θ, *b*) is the exact joint posterior. The usual method for performing this minimization is coordinate descent, i.e., one of the *q*'s is optimized at a time, with the others held fixed. The Infer.NET software program (http://research.microsoft.com/en-us/um/cambridge/projects/infernet/, Minka et al., 2012) provides several options for performing this minimization. In the current study, the bound of Saul and Jordan (1999) on the logistic function gave the best trade-off of speed vs. accuracy.

In the case of hierarchical priors, additional unknowns such as the means and precisions of the ability, discrimination (for 2-PL), and difficulty parameters (*m*_θ_, *u*_θ_, *m*_*a*_, *u*_*a*_, *m*_*b*_, and *u*_*b*_, respectively) must be dealt with. For their posterior distributions, a fully factorized approximation was used as follows for the 1-PL:

(6) $$q(m_{\theta },\;u_{\theta },\;m_{b},\;u_{b})=q(m_{\theta })q\left(u_{\theta }\right)q\left(m_{b}\right)q(u_{b})$$

where *q* was Gaussian for the *m*'s and Gamma for the *u*'s. The approximation for the 2-PL was:

(7) $$\begin{array}{l} q(m_{\theta },\;u_{\theta },\;m_{a},\;u_{a},m_{b},\;u_{b})=q(m_{\theta })q\left(u_{\theta }\right)q\left(m_{a}\right)q\left(u_{a}\right)\\ \;q\left(m_{b}\right)q(u_{b}). \end{array}$$

The KL divergence is formed over the larger set of unknowns and minimized as before. Because these new distributions have two free parameters each, there are 8 and 12 additional free parameters in the optimization process for 1-PL and 2-PL models, respectively.

## CML estimation

CML takes advantage of the sufficiency property of Rasch models: that the sum of each response vector is a sufficient statistic (Andersen, 1970). Therefore, ability estimates are not needed if conditioning is performed on raw scores, once extreme (0 or perfect) scores are removed. In this study, CML estimation was performed via the eRm (extended Rasch models; Mair and Hatzinger, 2007a,b; Mair et al., 2012) package in R (R Core Team, 2013). The difficulty parameters were normalized using the sum-to-zero option. After difficulty parameters were estimated, person parameters were estimated via ML using the CML difficulty estimates. Because the sufficiency property holds only for 1-PL, CML estimation was used only for the 1-PL model.

## MML estimation

In MML estimation item parameters are estimated by integrating the likelihood function with respect to the person parameter distribution (normal distribution) and maximizing the likelihood with respect to the item parameters (Bock and Aitkin, 1981). In contrast to CML, MML can include extreme score patterns, yet needs to make a distributional assumption about the person parameters. Because of the intractable nature of the likelihood function, numerical methods are utilized for integrating and maximizing the likelihood function. BILOG-MG (Zimowski et al., 2003) is a widely used software program for IRT estimation of item and examinee parameters. For more information on and how to use BILOG-MG, readers may refer to Rupp (2003). In this study, BILOG-MG was used with the option of estimating the item parameters using MML and ability parameters using the maximum likelihood (ML) method^2^.

## Methods

The primary purpose of this study was to investigate the impact of prior choice on 1-PL and 2-PL model parameter estimation for two Bayesian methods: MCMC and VB. Three prior choices were considered for each of the two Bayesian techniques.

### Matched prior

Matched prior refers to the same distribution that was used to simulate data. This may not be realistic, but is included as a gold-standard against which the other prior results were compared. The matched priors were: θ, *b* ~ *N*(0, 1) and *a*_*k*_ ~ lognormal(0, 0.25).

### Standard vague prior

This case refers to a situation where there is a large uncertainty in selecting the prior distribution. The degree of uncertainty is reflected in the variance of the prior distribution. In this study, the prior distribution for the ability parameter was set to the standard normal, while the prior for difficulty and discrimination parameters were modeled with large variances representing the most “pessimistic” belief that almost nothing is known about the parameter: θ ~ *N*(0, 1), *b* ~ *N*(0, 10^3^), *a*_*k*_ ~ lognormal(0, 8).

### Hierarchical prior

In this case, the parameters of the prior distributions are treated as random variables and given hyper-priors, which are vague. Table 1 below summarizes these priors for both Bayesian estimation methods. A relatively informative inverse gamma (1, 1) distribution was used for variance because Gelman (2006) cautioned against the use of very low values such as 0.01 and 0.001 for the gamma prior which lead to improper posteriors.

**Table 1:** **Estimation methods and prior distributions for normal data**.

**Estimation method**	**Prior choice**	**Prior distributions**	**Name**
MCMC	Matched	θ, *b* ~ normal(0, 1)	MCMC-matched
		a ~ lognormal(0, 0.25)	
	Standard vague	θ ~ normal(0, 1)	MCMC-stdvague
		*b* ~ normal(0, 10^3^)	
		*a* ~ lognormal(0, 8)	
	Hierarchical	θ ~ normal(*m*_θ_, ${\text{u}}_{\theta }^{-1}$)	MCMC-hierarchical
		*b* ~ normal(*m*_*b*_, ${\text{u}}_{\text{b}}^{-1}$)	
		*m*_θ_, *m_b_* ~ normal(0, 10^6^)	
		*u*_θ_, *u*_*b*_ ~ gamma(1, 1)	
		*a* ~ lognormal(*m*_*a*_, ${\text{u}}_{\text{a}}^{-1}$)	
		*m*_*a*_ ~ normal(0, 10^6^)	
		*u*_*a*_ ~ gamma(1, 1)	
Variational	Matched	Same as MCMC	VB-matched
Bayes	Standard vague	Same as MCMC	VB-stdvague
	Hierarchical	Same as MCMC	VB-hierarchical
CML^a^	NA		CML
MML^a^	NA		MML

a *CML and MML estimation methods were used for 1-PL data and only MML was used for 2-PL data*.

CML and MML estimates were used for baseline comparisons. Both the MML and CML methods were used for 1-PL data; only MML was used for 2-PL data. Other factors varied were sample size (250, 500, 1000, 2000) and test length (10, 20, 40). Sample size (4), and test length (3) were completely crossed, resulting in 12 conditions. For 1-PL data 8 estimation methods were completely crossed with the sample size and test length resulting in 96 conditions, whereas for 2-PL data 7 estimation methods were completely crossed with the sample size and test length resulting in 84 conditions.

## Data generation

Examinee abilities and item difficulties were generated from the standard normal distribution with mean 0 and standard deviation 1; item discrimination parameters were generated from the lognormal distribution with mean 0 and standard deviation 0.25. For generating 1-PL data, the probability of correct response was computed by fixing the discrimination parameter in Equation 1 to 1, and converting into a response of 0 or 1 using a randomly generated threshold from the uniform distribution. For the 2-PL data, the probability of correct response was computed using Equation 1, and converted into a response of 0 or 1 using a randomly generated threshold from the uniform distribution. For each condition, 100 data sets were simulated using MATLAB 7.11 (MATLAB, 2011).

Six different Bayesian estimations and one or two non-Bayesian estimations (MML and CML for 1-PL data and MML for 2-PL data) were performed on each data set. For each estimation method for 1-PL data, examinee ability and item difficulty parameters were estimated along with their respective standard errors (SEs). The probability of correct response for each examinee on each item was computed using the estimated parameters. For 2-PL data, the ability, difficulty, and discrimination parameters along with their respective SEs were estimated, and the probability of correct response for each examinee on each item was computed using the estimated parameters.

MCMC estimates were obtained using OpenBUGS (Lunn et al., 2009); VB estimates were obtained using Infer.Net (Minka et al., 2012). CML estimates were obtained via the eRm package in R and MML estimates were obtained via BILOG-MG.

For the MCMC analysis, the first 5000 samples were discarded (burn-in) and the next 1000 samples were used for parameter estimation. The mean of the posterior distribution was taken as the value for each estimate. In order to speed convergence, the initial values of the means were set at 0 for both standard vague and hierarchical priors, while the initial values of the standard deviations were set at 1 for hierarchical priors. Adequacy of convergence of the parameter estimates was checked using convergence diagnostics produced by the Bayesian output analysis (BOA; Smith, 2007) program. BOA generates several diagnostic indices, of which three were considered: multivariate potential scale reduction factor (MPSRF; Brooks and Gelman, 1998), estimated potential scale reduction (EPSR; Gelman, 1996), and the half-width test (Heidelberger and Welch, 1983). Convergence is indicated when the 0.975th quantiles of both scale reduction factors are < 1.2, whereas for the half-width test the choice is pass/fail. Results for all conditions met all convergence criteria, indicating that the samples were drawn from fairly stationary distributions. However, this does not imply that the number of samples was always adequate for accurate estimation of parameters. In some cases with the hierarchical prior, it appears that 1000 samples may not have been enough to match the accuracy of the variational approximation.

## Evaluation criteria

The average root mean squared error (RMSE) of the ability parameter for a given sample of *S* examinees was computed as:

(8) $$\begin{array}{l} \text{Average RMSE}\left(\theta \right)=\frac{1}{R}\sum_{r=1}^{R}\sqrt{\frac{1}{S}\sum_{s=1}^{S}{\left({\hat{\theta }}_{sr}-\theta_{sr}\right)}^{2}}, \end{array}$$

where ${\hat{\theta }}_{sr}$ and θ_*sr*_ were the estimated and real values of the ability parameter for replication *r* and examinee *s*, respectively. The average RMSE of the difficulty parameter for a given test length *L* was computed as:

(9) $$\begin{array}{l} \text{Average RMSE}\left(b\right)=\frac{1}{R}\sum_{r=1}^{R}\sqrt{\frac{1}{L}\sum_{i=1}^{L}{\left({\overset{⌢}{b}}_{ir}-\;b_{ir}\right)}^{2}}, \end{array}$$

where ${\overset{⌢}{b}}_{ir}$ and *b*_*ir*_ were the estimated and real values of the difficulty parameter for replication *r* and item *i*, respectively, and *R* is the total number of replications (in this case, 100). The average RMSE of the discrimination parameter for a given test length *L* was computed as:

(10) $$\begin{array}{l} \text{Average RMSE}\left(a\right)=\frac{1}{R}\sum_{r=1}^{R}\sqrt{\frac{1}{L}\sum_{i=1}^{L}{\left({â}_{ir}-\;a_{ir}\right)}^{2}}, \end{array}$$

where â_*ir*_ and *a*_*ir*_ were the estimated and real values of the discrimination parameter for replication *r* and item *i*, respectively, and *R* is the total number of replications (in this case, 100).

The 2-PL model has a shift and scale ambiguity in the parameters, in the sense that (θ, *b, a*) can be transformed into an equivalent (θ′, *b*′, *a*′) given by Equations (11–13) for any *s, t*.

(11) $$\begin{array}{rcl} a_{k}^{\prime } & = & \frac{a_{k}}{s} \end{array}$$

(12) $$\begin{array}{rcl} b_{k}^{\prime } & = & b_{k}s+t \end{array}$$

(13) $$\begin{array}{rcl} \theta_{i}^{\prime } & = & \theta_{i}s+t \end{array}$$

In order to measure the RMSE fairly, we took the estimates from each method in each trial and transformed them as above in order to minimize the RMSE to the true values. That is, (*s, t*) were chosen to minimize

(14) $$\begin{array}{l} J\left(s,\;t\right)=RMSE\left(\theta |s,t\right)+RMSE\left(b|s,t\right)+RMSE\left(a|s,\;t\right), \end{array}$$

where *RMSE*(θ|*s, t*) is the RMSE formula in Equation (8) where the estimated θ is transformed by (*s, t*).

Accuracy of parameter estimates was evaluated using the RMSE between the real (simulated) and shifted values of the estimated parameters. Note this is more accurate than simply computing the correlation between the estimated and real parameters since a correlation allows each parameter to have its own shift and scale, which is not consistent with the model.

Even with these adjustments, looking at the RMSE of individual parameters can be misleading and inconclusive. For example, if method A has low RMSE for difficulty while method B has low RMSE for ability, which should we regard as better? For this reason, we also include RMSEs for the probabilities of correct responses. This provides a single direct measure of how well the model has fit the data. The RMSEs were averaged over all 100 replications. The average RMSE of the probability of correct response for a given test length *L* and sample size *S* was computed as:

(15) $$\begin{array}{l} \text{Average RMSE}\left(p\right)=\frac{1}{R}\sum_{r=1}^{R}\sqrt{\frac{1}{LS}\sum_{s=1}^{S}\sum_{i=1}^{L}{\left({\overset{⌢}{p}}_{isr}-\;p_{isr}\right)}^{2}}, \end{array}$$

where ${\overset{⌢}{p}}_{isr}$ and *p*_*isr*_ were the probabilities of examinee *s* replication *r* and item *i* computed based on estimated and real parameters, respectively. Finally, RMSEs were evaluated based on comparison (i.e., whether one condition had lower RMSE than another) and not based on any absolute cut-off.

In order to compare the accuracy of parameter estimates with respect to the estimation method (EM), sample size (SS), test length (TL), and all possible interactions, five separate analyses of variance (ANOVA) were conducted for 1-PL data and seven separate analyses of variance (ANOVA) were conducted for 2-PL data. The five dependent variables corresponding to the five ANOVAs for the 1-PL data were: average RMSEs for difficulty, ability, and probability estimates, and average SEs for ability and difficulty estimates. For the 2-PL data, the two additional ANOVAs included average RMSEs and average SEs for the discrimination parameter estimates. For each ANOVA the same independent variables were utilized: EM (eight levels for 1-PL and seven levels for 2-PL), SS (four levels), and TL (three levels) and all possible interactions.

For each ANOVA, effect sizes (η^2^) were used to assess the effect of each independent variable and two way interactions on the respective estimate. Following Cohen (1988), an effect size greater than 14% was considered large, between 8 and 14% medium, and below 8% small.

## Results

### 1-PL data

ANOVA results for the 1-PL data conditions are shown in Table 2; the corresponding average RMSEs and SEs are shown in Table 3. Table 2 shows the percentage of variance explained (η^2^) for both main and interaction effects that explain at least 8% of variance. It can be seen that the estimation method (EM) explains the majority of the total variance in the RMSEs for ability, difficulty, and the probability of correct response estimates. EM also explains a major portion of the variance for the SEs of the difficulty parameter. The TL on the other hand explains major portion of the variance for the ability SEs.

**Table 2:** **Effect sizes (η^2^) from ANOVA for 1-PL data in percentages**.

**Effect**	**Average RMSE**			**Average SE**	
	**Ability**	**Difficulty**	**Probability**	**Ability**	**Difficulty**
EM	84.413	99.327	95.163	18.014	60.936
TL	11.484			72.505	
SS					21.736
EM × SS					9.436

**Table 3:** **Marginal means by estimation method for 1-PL data**.

**Estimation method**	**Ability**		**Difficulty**		**Probability**
	**RMSE**	**SE**	**RMSE**	**SE**	**RMSE**
CML	0.60	0.58	0.09	0.09	0.11
MCMC-hierarchical	0.47	0.60	0.09	0.35	0.09
MCMC-matched	0.47	0.47	0.09	0.10	0.09
MCMC-stdvague	1.33	0.48	1.40	0.11	0.36
MML	0.60	0.59	0.10	0.10	0.16
VB-hierarchical	0.47	0.46	0.09	0.09	0.09
VB-matched	0.47	0.46	0.09	0.09	0.09
VB-stdvague	0.47	0.46	0.10	0.10	0.09

#### Ability estimate

Table 2 shows that EM accounts for 84% of the variance in the average RMSEs of the ability estimate, but only 18% of the variance in SEs of ability estimates. In contrast, TL accounted for 72% of the variance in SEs and only 11% in EM. It can be seen from Table 3 that all Bayesian estimates, with the exception of MCMC-standard vague, have the lowest RMSEs. For the SEs, all Bayesian estimates, with the exception of MCMC-hierarchical, have the lowest values. As expected, increasing test length was associated with a decrease in both the RMSEs and SEs. This was true across all estimation methods.

#### Difficulty estimate

Table 2 shows that EM explains 99% of the variance in the average RMSEs of the difficulty estimate, and about 61% of the variance in SEs. Sample size and the interaction between sample size and EM explain about 22 and 9.5% of the variance in the difficulty SEs, respectively. As can be seen from Table 3, all estimation methods have lower and similar RMSEs than MCMC-standard vague values. For SEs, all estimation methods, except MCMC-hierarchical, have lower and similar values. The MCMC-hierarchical SEs are more than three times larger than other methods. Marginal means aggregated over test length and sample size are given in Table 4.

**Table 4:** **Marginal means aggregated over test length and sample size for the 1PL model**.

	**SS**	**CML**	**MML**	**MCMC**			**VB**		
				**Hierarchical**	**Matched**	**Std_vague**	**Hierarchical**	**Matched**	**Std_vague**
diff-RMSE	250	0.15	0.16	0.15	0.15	1.39	0.15	0.15	0.15
	500	0.10	0.11	0.10	0.10	1.39	0.10	0.10	0.10
	1000	0.07	0.08	0.07	0.07	1.40	0.07	0.07	0.07
	2000	0.05	0.06	0.05	0.05	1.40	0.05	0.05	0.05
diff-SE	250	0.15	0.16	0.55	0.16	0.16	0.15	0.15	0.15
	500	0.10	0.11	0.38	0.11	0.12	0.10	0.10	0.10
	1000	0.07	0.08	0.27	0.08	0.08	0.07	0.07	0.07
	2000	0.05	0.06	0.19	0.06	0.06	0.05	0.05	0.05
	**TL**								
ab-RMSE	10	0.82	0.82	0.60	0.60	1.28	0.60	0.60	0.60
	20	0.58	0.58	0.47	0.47	1.33	0.47	0.47	0.47
	40	0.40	0.40	0.35	0.35	1.37	0.35	0.35	0.35
ab-SE	10	0.79	0.83	0.77	0.60	0.60	0.58	0.59	0.59
	20	0.56	0.57	0.59	0.47	0.47	0.46	0.46	0.46
	40	0.39	0.39	0.44	0.35	0.35	0.35	0.35	0.35
p-RMSE	10	0.14	0.19	0.11	0.11	0.36	0.11	0.11	0.11
	20	0.10	0.15	0.10	0.09	0.36	0.09	0.09	0.09
	40	0.07	0.13	0.07	0.07	0.37	0.07	0.07	0.07

#### Probability estimate

To examine the concurrent effect of both ability and difficulty estimates and their SEs, probabilities of correct responses were computed and their SEs were averaged. Examining these values from Table 3, it can be seen that all Bayesian estimation methods with the exception of MCMC-standard vague have lower values than both CML and MML SEs.

### 2-PL data

MCMC standard vague did not converge for some datasets when using OpenBUGS. Therefore, we implemented our own MCMC algorithm to estimate the 2-PL parameters. VB standard vague also did not converge in several conditions, but improvements to this algorithm could not be made. Therefore, VB standard vague results are not reported. ANOVA results for the 2-PL data are shown in Table 5. It shows effects (main and interaction) that explain at least 8% of variance (η^2^) in average RMSEs and average SEs. For the average RMSEs, TL explains the majority of variance for ability and probability estimates, while EM explains the majority of variance for the difficulty estimate; SS explains the majority of variance for the discrimination parameter estimate. For the average SEs, EM explains the majority of variance for all three parameters, although both TL and SS play a significant role.

**Table 5:** **Effect sizes (η^2^) from ANOVA for 2-PL data in percentages**.

**Effect**	**Average RMSE**				**Average SE**		
	**Ability**	**Difficulty**	**Discrimination**	**Probability**	**Ability**	**Difficulty**	**Discrimination**
EM		56.32	16.58		50.18	48.92	42.51
TL	93.19			88.36	26.15		
SS		21.61	47.19			22.05	19.73
EM × SS		21.22	25.69			22.14	16.95

#### Ability estimate

Table 5 shows that TL accounts for 93% of the variance in the average RMSEs of the ability estimate, while for average ability SEs both EM and TL account for 50 and 26% of the variance, respectively. Table 6 shows marginal means of average RMSEs aggregated over SS. Table 7 shows marginal means aggregated over TL and SS. From Table 6 it can be seen that both RMSEs and SEs of the ability estimate decrease as the TL increases. This is true for all estimation methods and all prior choices. For TL of 10 items, all estimation methods have similar RMSEs; however, as the TL increases to 20 and 40 items, Bayesian estimates have similar and smaller values than MML RMSEs. For SEs, hierarchical priors have the highest SEs, while the rest of the Bayesian SEs are lower than MML SEs. From Table 8 it can be seen that ability SEs are lowest for VB-matched, MCMC-matched, and MCMC-standard vague.

**Table 6:** **Marginal means aggregated over sample size for the 2-PL model^a^**.

**Parameter**	**Test length**	**MML**	**MCMC**			**VB**	
			**Matched**	**Standard vague**	**Hierarchical**	**Hierarchical**	**Matched**
Ability RMSE	10	0.61	0.59	0.60	0.61	0.59	0.59
	20	0.51	0.46	0.47	0.48	0.46	0.46
	40	0.46	0.35	0.35	0.36	0.35	0.35
Ability SE	10	0.82	0.59	0.60	1.37	0.98	0.58
	20	0.56	0.47	0.48	1.00	0.80	0.45
	40	0.38	0.35	0.37	0.69	0.63	0.34
Probability RMSE	10	0.14	0.12	0.12	0.12	0.12	0.11
	20	0.10	0.09	0.09	0.09	0.09	0.09
	40	0.08	0.07	0.07	0.07	0.07	0.07

a *For the 2-PL data, VB-stdvague estimates did not converge for several conditions. Therefore they are not reported*.

**Table 7:** **Marginal means aggregated over test length for the 2PL model**.

**Parameter**	**Sample size**	**MML**	**MCMC**			**VB**	
			**Matched**	**Standard vague**	**Hierarchical**	**Hierarchical**	**Matched**
Difficulty RMSE	250	0.41	0.18	0.6	0.26	0.19	0.18
	500	0.4	0.14	0.27	0.19	0.14	0.14
	1000	0.4	0.1	0.13	0.13	0.11	0.1
	2000	0.4	0.08	0.09	0.09	0.08	0.08
Difficulty SE	250	0.31	0.2	1.18	1.27	0.26	0.15
	500	0.21	0.15	0.35	0.92	0.19	0.11
	1000	0.13	0.11	0.15	0.58	0.14	0.08
	2000	0.09	0.08	0.1	0.38	0.1	0.05
Discrimination RMSE	250	0.23	0.16	0.46	0.19	0.17	0.16
	500	0.16	0.13	0.19	0.14	0.13	0.13
	1000	0.11	0.1	0.12	0.11	0.1	0.1
	2000	0.08	0.07	0.08	0.08	0.07	0.08
Discrimination SE	250	0.15	0.17	0.5	0.35	0.07	0.12
	500	0.1	0.14	0.19	0.29	0.05	0.09
	1000	0.07	0.11	0.12	0.25	0.04	0.06
	2000	0.05	0.08	0.08	0.2	0.03	0.05

**Table 8:** **Marginal means aggregated over SS and TL for the 2PL model**.

**Parameter**	**Statistic**	**MML**	**MCMC**			**VB**	
			**Matched**	**Standard vague**	**Hierarchical**	**Matched**	**Hierarchical**
Ability	RMSE	0.52	0.47	0.47	0.48	0.47	0.47
	SE	0.59	0.47	0.48	1.02	0.46	0.8
Difficulty	RMSE	0.4	0.13	0.27	0.17	0.13	0.13
	SE	0.18	0.14	0.44	0.79	0.1	0.17
Discrimination	RMSE	0.15	0.12	0.21	0.13	0.12	0.12
	SE	0.09	0.12	0.22	0.27	0.08	0.05
Probability	RMSE	0.11	0.09	0.09	0.09	0.09	0.09

#### Difficulty estimate

Table 5 shows that both average RMSEs and average SEs for the difficulty estimate are affected by EM, SS, and the interaction of EM and SS. The EM accounts for about 50% or more of the variance for both RMSEs and SEs. Table 7 shows results across SS and Table 8 shows results across EM methods. From Table 8 it can be seen that both RMSEs and SEs are lowest for MCMC-matched, VB-matched, and VB-hierarchical. From Table 7 it can be seen that the average RMSEs decrease with an increase in sample size for all estimation methods except for MML. The error decreases by more than half as the SS increases from 250 to 2000. For MML, the average error is not affected by the SS. The average SEs decreases with increasing SS for all estimation methods without exception.

#### Discrimination estimate

Similar to the difficulty parameter, the discrimination parameter also is affected by EM, SS, and the interaction of EM and SS. For the RMSEs, a substantial portion of the variance (47%) is accounted for by the SS, while for the SEs, a substantial portion of the variance (43%) is accounted for by the EM. From Table 7 it can be seen that as the SS increases from 250 to 2000, RMSEs drastically decrease for all estimation methods. While the same trend can be observed for the SEs also, the decrease in the SEs is not as steep as the decrease in RMSEs. From Table 8 it can be observed that for RMSEs, all Bayesian methods, except the MCMC-standard vague, have lower values than MML; for SEs, MCMC-standard vague and MCMC-hierarchical have the highest values and VB-matched and VB-hierarchical have the lowest values.

#### Probability estimate

Probability of correct response, which takes into account both ability and item estimates, provides an overall effect of the estimates on the item responses. It can be seen from Table 8 that RMSEs associated for the probability of correct response are about the same for all the Bayesian estimates and are lower than RMSEs of MML. Similar to the ability estimate, RMSEs for the probability of correct response is only affected by the TL, accounting for 88% of the variance. From Table 6 it can be seen that as the TL increases from 10 to 40, the RMSE decreases by about half for all estimation methods.

## Summary and conclusion

Through statistical simulation, the present study showed that practitioners would benefit from using Variational Bayesian with hierarchical priors to estimate parameters of 1-PL and 2-PL models. This method is cost-effective, quick, and accurate. Infer.NET is a freely available software program that implements VB. The codes for 1-PL and 2-PL estimation are available upon request. The hierarchical priors used in the present study covered a far wider range of item and person parameter values than those encountered in psychometric research. Therefore, users may apply these programs to any dataset they would use for 1-PL and 2-PL applications.

EM, TL, and SS played significant roles in accurately estimating item and examinee parameters. This reflects the findings of Kieftenbeld and Natesan (2012), Wollack et al. (2002), Swaminathan and Gifford (1982), and Roberts and Thompson (2011) who all found that test length affected the accuracy of examinee parameters and sample size affected the accuracy of item parameters. With respect to the sample size, and RMSEs our results were similar to Sheng's (2010) with one exception. In our study the average RMSEs decreased as the sample size increased for both difficulty and discrimination parameters, whereas in Sheng's RMSEs decreased with increased sample size only for the discrimination parameter. Regarding the test length our results were similar to Sheng (2010), namely, the test length had no effect in estimating discrimination and difficulty parameters. Standard vague priors produced relatively large RMSEs and in some cases did not converge. This confirms Sheng's (2010) suggestions about not using extremely vague priors to avoid convergence issues. The RMSEs were lower for all Bayesian methods (with the exception of standard vague priors) than the CML and MML estimates. SEs for VB estimation methods (with the exception of standard vague priors) were either lowest or about the same as other methods for all conditions except one case (2PL ability estimates). Surprisingly, the MCMC-hierarchical SEs were, most of the time, larger than other methods. With a few exceptions hierarchical priors showed superior performance. Matched and hierarchical produced comparable results. Overall, VB-hierarchical, VB-matched, and MCMC-matched performed uniformly well in most situations and produced the lowest RMSEs and SEs in most cases.

Although the matched prior results were very similar to the hierarchical priors, such matched priors are unavailable to practitioners in real data applications because the actual distribution of parameters is unknown. Therefore, researchers must be cautious about choosing either extremely informative or extremely vague priors when the true parameter distribution is unknown. Unless previous research provides convincing evidence about parameter distributions, very informative or vague priors should be avoided in practice. Because hierarchical priors produced very similar results in this simulation where the true distributions known, hierarchical priors appear advantageous and are recommended for both MCMC and VB estimation in practice.

Additionally, this study has demonstrated that VB is a viable alternative to MCMC for the estimation of model parameters in a Bayesian framework. The results showed that in most cases there is no loss in accuracy of parameter estimates in VB, while gaining the efficiencies of faster estimation, making it an attractive choice for Bayesian estimation. However, the trade-off could be some loss in precision as evidenced in the case of SEs of ability estimates for 2-PL data. VB-hierarchical priors recovered the parameters as well as matched priors did, even for small samples. The savings in computational time could be significant for VB. For example, OpenBUGS took 336 s to estimate the parameters of 2000 examinees and 40 items using standard priors while VB took 8.62 s and BILOG-MG took 1.5 s. OpenBUGS estimation time increased (5 h for hierarchical priors), while that of VB decreased (6.5 s) for complex prior specifications. Our own Gibbs sampler with hierarchical priors took 102.09 s for estimation. It is also possible that other Bayesian programs such as just another Gibbs sampler (JAGS, http://mcmc-jags.sourceforge.net/) and STAN (http://mc-stan.org/) are more computationally efficient than OpenBUGS.

Future research needs to focus on more complex models such as the 3-PL and polytomous models and under varied conditions to further investigate the viability of VB as a reliable estimation method. Further research also needs to be done on how reliable prior information can be used for parameter recovery in small samples using both VB and MCMC. In the present study OpenBUGS ran into convergence issues for vague priors for the 2-PL model. In such instances, the researcher may have to find alternate solutions such as writing their own Gibbs samplers. Therefore, there is need for research that focuses specifically on developing and making available algorithms for IRT models.

With VB, we now have the opportunity to exploit the advantages of Bayesian estimation (i.e., estimating complex models, incorporating information from previous studies, and placing realistic constraints on parameters) while liberating ourselves from the disadvantages of MCMC (i.e., non-convergence and time-intensive computation). The possibility of expanding the use of VB for more complex models than 1-PL and 2-PL may encourage the measurement field to investigate newer item response models and to operationalize Bayesian estimation into more mainstream operational examinations^3^.

## Author contributions

PN initiated the paper, simulated the data, ran the MCMC estimation, wrote the relevant sections, and coordinated team meetings. RN advised on simulation conditions and model choice, oversaw MML estimation, and wrote the relevant sections. TM advised on simulation diagnostics, wrote and ran VB estimation, aggregated simulation results, and wrote the relevant sections. JR advised on relevance of the models to the testing industry, ran the MML estimation, and wrote the relevant sections.

### Conflict of interest statement

The authors declare that the research was conducted in the absence of any commercial or financial relationships that could be construed as a potential conflict of interest.

## Footnotes

^1^Local independence refers to the condition where responses to items are independent for a given level of ability. That is, when the ability level is held constant, the probability of responding to any item is independent of other items.

^2^This option was selected (as opposed to EAP estimation) so that it utilizes purely non-Bayesian estimation.

^3^VB program for 1-PL and 2-PL is available upon request.

## References

[B1] Andersen E. B. (1970). Asymptotic properties of conditional maximum likelihood estimators. J. R. Stat. Soc. B 32, 283–301.

[B2] Ansari A. Jedidi K. (2000). Bayesian factor analysis for multilevel binary observations. Psychometrika 65, 475–496. 10.1007/BF02296339

[B3] Baker F. B. Kim S. (2004). Item Response Theory: Parameter Estimation Techniques, 2nd Edn. New York, NY: Taylor and Francis.

[B4] Beal M. J. Ghahramani Z. (2003). The variational Bayesian EM algorithm for incomplete data: with application to scoring graphical model structures, in Bayesian Statistics, Vol. 7, eds Bernardo J. M. Bayarri M. J. Berger J. O. David A. P. Heckerman D. Smith A. F. M. West M. (Oxford, UK: Oxford University Press), 453–464.

[B5] Bishop C. M. (2006). Pattern Recognition and Machine Learning. New York, NY: Springer.

[B6] Bock R. D. Aitkin M. (1981). Marginal maximum likelihood estimation of item parameters: an application of an EM algorithm. Psychometrika 46, 443–459. 10.1007/BF02293801

[B7] Brooks S. P. Gelman A. (1998). General methods for monitoring convergence of iterative simulations. J. Comput. Graph. Stat. 7, 434–455.

[B8] Cohen J. (1988). Statistical Power Analysis for the Behavioral Sciences, 2nd Edn. Hillsdale, NJ: Erlbaum.

[B9] Fox J.-P. (2010). Bayesian Item Response Modeling: Theory and Applications. New York, NY: Springer.

[B10] Gao F. Chen L. (2005). Bayesian or non-Bayesian: a comparison study of item parameter estimation in the three-parameter logistic model. Appl. Meas. Educ. 18, 351–380. 10.1207/s15324818ame1804_227512826

[B11] Gelfand A. E. Smith A. F. M. (1990). Sampling-based approaches to calculating marginal densities. J. Am. Stat. Assoc. 85, 398–409. 10.1080/01621459.1990.10476213

[B12] Gelman A. (1996). Inference and monitoring convergence, in Markov Chain Monte Carlo in Practice, eds Gilks W. R. Richardson S. Spiegelhalter D. J. (London: Chapman and Hall), 131–144.

[B13] Gelman A. (2006). Prior distributions for variance parameters in hierarchical models. Bayesian Anal. 3, 515–533. 10.1214/06-BA117A

[B14] Gelman A. Carlin J. B. Stern H. S. Dunson D. B. Vehtari A. Rubin D. B. (2013). Bayesian Data Analysis, 3rd Edn. London: Chapman and Hall.

[B15] Gelman A. Jakulin A. Pittau M. G. Su Y.-S. (2008). A weakly informative default prior distribution for logistic and other regression models. Ann. Appl. Stat. 2, 1360–1383. 10.1214/08-AOAS191

[B16] Geman S. Geman D. (1984). Stochastic relaxation, Gibbs distribution and Bayesian restoration of images. IEEE Trans. Pattern Anal. Mach. Intell. 6, 721–741. 10.1109/TPAMI.1984.4767596 22499653

[B17] Ghosh M. Ghosh A. Chen M. Agresti A. (2000). Bayesian estimation for item response models. J. Stat. Plan. Inference 88, 99–115. 10.1016/S0378-3758(99)00201-3

[B18] Heidelberger P. Welch P. D. (1983). Simulation run length control in the presence of an initial transient. Oper. Res. 31, 1109–1144. 10.1287/opre.31.6.1109

[B19] Kieftenbeld V. Natesan P. (2012). Recovery of graded response model parameters: a comparison of marginal maximum likelihood and Markov chain Monte Carlo estimation. Appl. Psychol. Meas. 36, 399–419. 10.1177/0146621612446170

[B20] Kim S. (2001). An evaluation of the Markov chain Monte Carlo method for the Rasch model. Appl. Psychol. Meas. 25, 163–176. 10.1177/01466210122031984

[B21] Kullback S. Leibler R. A. (1951). On information and sufficiency. Ann. Math. Stat. 22, 79–86. 10.1214/aoms/1177729694

[B22] Lord F. M. (1986). Maximum likelihood and Bayesian parameter estimation in item response theory. J. Educ. Meas. 23, 157–162. 10.1111/j.1745-3984.1986.tb00241.x

[B23] Lunn D. Spiegelhalter D. Thomas A. Best N. (2009). The bugs project: evolution, critique and future directions. Stat. Med. 28, 3049–3082. 10.1002/sim.3680 19630097

[B24] Mair P. Hatzinger R. (2007a). Extended Rasch modeling: the eRm package for the application of IRT models in R. J. Stat. Softw. 20, 1–20. 10.18637/jss.v020.i09

[B25] Mair P. Hatzinger R. (2007b). CML based estimation of extended Rasch models with the eRm package in R. Psychol. Sci. 49, 26–43.

[B26] Mair P. Hatzinger R. Maier M. J. (2012). eRm: Extended Rasch Modeling. R Package Version 0.15-1. Available online at: http://CRAN.R-project.org/package=eRm

[B27] MATLAB (2011). MATLAB and Statistics Toolbox Release, 2011, MATLAB 7.11. Natick, MA: The MathWorks, Inc.

[B28] Minka T. Winn J. Guiver J. Knowles D. (2012). Infer.NET 2.5 [Software]. Cambridge, UK: Microsoft Research Cambridge.

[B29] Patz R. J. Junker B. W. (1999). A straightforward approach to Markov Chain Monte Carlo methods in item response models. J. Educ. Behav. Stat. 24, 146–178. 10.2307/1165199

[B30] R Core Team (2013). R: A Language and Environment for Statistical Computing. Vienna: R Foundation for Statistical Computing.

[B31] Roberts J. S. Thompson V. M. (2011). Marginal maximum a posteriori item parameter estimation for the generalized graded unfolding model. Appl. Psychol. Meas. 35, 259–279. 10.1177/0146621610392565

[B32] Rupp A. A. (2003). Item response modeling with BILOG-MG and MULTILOG for Windows. Int. J. Test. 3, 365–384. 10.1207/S15327574IJT0304_5

[B33] Saul L. Jordan M. (1999). A mean field learning algorithm for unsupervised neural networks, in Learning in Graphical Models, ed Jordan M. I. (Cambridge, MA: MIT Press), 541–554.

[B34] Sheng Y. (2010). A sensitivity analysis of Gibbs sampling for 3PNO IRT models: effects of prior specifications on parameter estimates. Behaviormetrika 37, 87–110. 10.2333/bhmk.37.87

[B35] Smith B. J. (2007). boa: an R package for MCMC output convergence assessment and posterior inference. J. Stat. Softw. 21, 1–37. 10.18637/jss.v021.i11

[B36] Swaminathan H. Gifford J. (1982). Bayesian estimation in the Rasch model. J. Educ. Stat. 7, 175–191. 10.2307/1164643

[B37] Wollack J. A. Bolt D. M. Cohen A. S. Lee Y.-S. (2002). Recovery of item parameters in the nominal response model: a comparison of marginal maximum likelihood estimation and Markov chain Monte Carlo estimation. Appl. Psychol. Meas. 26, 337–350. 10.1177/0146621602026003007

[B38] Zimowski M. Muraki E. Mislevy R. Bock R. (2003). BILOG-MG 3 for Windows: Multiple-Group IRT Analysis and Test Maintenance for Binary Items. Lincolnwood, IL: Scientific Software International.

